# Recent progress on peripheral neural interface technology towards bioelectronic medicine

**DOI:** 10.1186/s42234-020-00059-z

**Published:** 2020-11-30

**Authors:** Youngjun Cho, Jaeu Park, Chengkuo Lee, Sanghoon Lee

**Affiliations:** 1 Daegu Geongbuk Institute of Science and Technology (DGIST), Daegu, 42899 Republic of Korea; 2 Electrical & Computer Engineering, National University of Singapore, Singapore, 117583 Singapore; 3 Center for Intelligent Sensors and MEMS (CISM), National University of Singapore, Singapore, 117608 Singapore; 4 NUS Graduate School for Integrated Science and Engineering (NGS), National University of Singapore, Singapore, 117456 Singapore

**Keywords:** Peripheral nerve interface, Wireless neural interface, Energy harvesters, Ultrasound stimulation, Magnetic stimulation, Bioelectronic medicine

## Abstract

Modulation of the peripheral nervous system (PNS) has a great potential for therapeutic intervention as well as restore bodily functions. Recent interest has focused on autonomic nerves, as they regulate extensive functions implicated in organ physiology, chronic disease state and appear tractable to targeted modulation of discrete nerve units. Therapeutic interventions based on specific bioelectronic neuromodulation depend on reliable neural interface to stimulate and record autonomic nerves. Furthermore, the function of stimulation and recording requires energy which should be delivered to the interface. Due to the physiological and anatomical challenges of autonomic nerves, various forms of this active neural interface need to be developed to achieve next generation of neural interface for bioelectronic medicine. In this article, we present an overview of the state-of-the-art for peripheral neural interface technology in relation to autonomic nerves. Also, we reveal the current status of wireless neural interface for peripheral nerve applications. Recent studies of a novel concept of self-sustainable neural interface without battery and electronic components are presented. Finally, the recent results of non-invasive stimulation such as ultrasound and magnetic stimulation are covered and the perspective of the future research direction is provided.

## Introduction

**Fig. 1:**
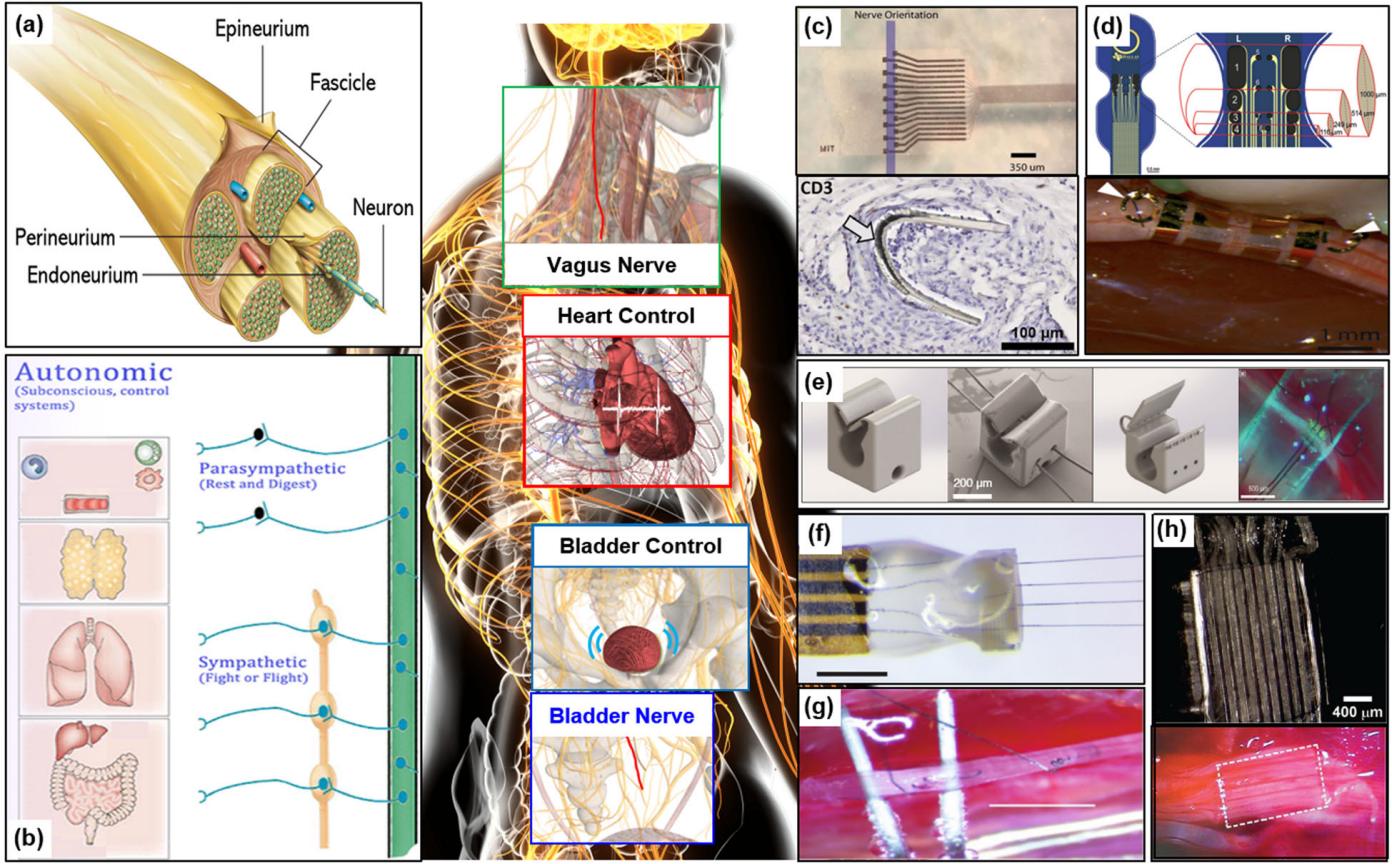
Several peripheral nerve interfaces to control the autonomic nervous system. **a** Anatomic structure and cross section of a peripheral nerve. **b** Schematic diagram of Autonomic Nervous System. **c** A novel flexible cuff-like microelectrode and the microscope image of the electrode encapsulated with tissue. **d** Schematic diagram of thin film multi-electrode and softening cuff implanted on a sciatic nerve. **e** Micro-scale printable nano-clips and the microscope picture of implanted nano-clip on a nerve. **f** Carbon fiber on polyimide ultra-microelectrodes. **g** Carbon nanotube (CNT) yarn electrodes. **h** microchannel electrodes for bladder control

With enormous progress on neural interface technology attributed to the development of cutting-edge technology of micro/nano material engineering with neuroscience, modulation of the autonomic nervous system (ANS) has recently emerged as a powerful way of modulating bodily functions and treating many diseases. Peripheral nervous system (PNS) has a cable-like structure, and extends from the spinal cord or brain, and starts branching functionally and separating further (Fig. 1a). Also, certain nerves contain afferent and efferent nerve fibers while others have only one type. Most nerves are located near muscles and organs, so approaching the nerves and recording neural signals from them are quite challenging. In particular, the autonomic nerves are very small, vulnerable for damage, and are associated with vital functions in the body (Fig. 1b). Nevertheless, modulation of some nerves is continuously targeted for therapeutic effects and is already shown reliable solutions for some diseases.

The extra-neural electrode which approaches by wrapping or attaching on the epineurium has the advantage of minimizing damage to the nerve, but it has the disadvantage that it has a lower signal-to-noise ratio (SNR) for a recording as well as lower resolution of stimulation than the intra-neural electrode which penetrate inside the epineurium. Since the limitation is originated from position of the electrodes, where it records or stimulates the nerve signal at the outer layer of a nerve, intra-neural approaches are considered depending on target nerves. However, autonomic nerves might be sensitive for penetration damage through the intra-neural approach in terms of anatomical and physiological aspects, so precise control supported by micro/nano engineering should be considered. Furthermore, biocompatibility and softness are critically important to issue during chronic implantation, such as immune responses that degrade the neural interface function. This issues can be result in interfering with chronic implantation.

The modulation of a nerve requires energy (Wang et al. 2020c Front. Comput. Neurosci). For instance, electrical stimulation involves delivering electrical pulses to a nerve, and neural recording needs to operate amplifiers to detect neural signals mixed with noises. With the development of neural interfaces for the autonomic nervous system, the method of delivering energy to the interface will also be a critical issue for the next generation of bioelectronic medicine.

## Autonomic nerve interface

Among the autonomic nerves, recent research on the vagus nerve has been actively investigated. The vagus nerve is an important tenth cranial nerve (CN X) that communicates between brain and organs, so various therapeutic interventions have been suggested and demonstrated by stimulating the vagus nerve. For instance, vagus nerve stimulation (VNS) was approved by the Food and Drug Administration (FDA) for patients with medically refractory partial onset seizures as an adjunctive, nonpharmacological therapy as well as for patients with chronic or recurrent major depression (O’Reardon et al. 2006). Furthermore, many researchers are focusing on other possible therapeutic modalities through VNS. The anti-inflammatory effect through VNS was demonstrated (Meregnani et al. 2011), and recently, the positive possibility of treating Crohn’s disease, rheumatoid arthritis, obesity, and diabetes, were reported (Bonaz et al. 2016; Koopman et al. 2016; de Lartigue 2016; Meyers et al. 2016).

One of the other applications targeting autonomic nerves is to modulate bladder function. The loss of bladder function caused by diseases or accidents is a critical issue for daily life of patients. For instance, spinal cord injury involves a serious complication with the loss of bladder function, and current approaches have limitations. An alternative approach is proposed through the modulation of bladder nerves by using neural interfaces (Chew et al. 2013). Also, many studies were conducted to resolve diseases such as bladder dysfunction and other bladder disorder (McGee et al. 2015).

In the early stages of research, studies are being conducted in mice or rats at the beginning to demonstrate the therapeutic potentials by modulating autonomic nervous system. However, the diameter of autonomic nerves in rodents are very small (100–300 μm), therefore, it is important to develop the neural interface that can interface (stimulation and recording) with small nerves for long-term (chronic) implantation.

Typically, biocompatible polymer materials such as polyimide, Parylene-C, and PDMS, etc. were used for the body of neural electrodes due to compatible micropatterning with microfabrication. Micropatterning using these materials has produced benefits such as selective recording and stimulation, easy implantation and stable recording through diversification of neural interface design (Fig. 1c-h) (Caravaca et al. 2017; Lee et al. 2015; Xiang et al. 2016; Lee et al. 2017a, b)**.**

The commercial cuff electrode using silicon has property of softness (1–50 MPa), but a thickness of 200 ~ 600 μm required for self-closing mechanism exacerbates fibrotic response. Recently, shape memory polymers (SMPs) were designed to operate a specific shape at body temperature, and studies on neural interfaces using SMP were demonstrated. An SMP neural interface was developed and implanted to the pelvic nerve (PN) and the vagus nerve (VN). That can be softened at 37 °C (550 MPa) with a thin thickness of 30 μm to minimize damage to nerves and neural interfaces (Fig. 1d) (González-González et al. 2018). Furthermore, a neural interface that can interface with small nerves with a diameter of about 50 μm and chronic implantation for up to 74 days to tracheosyringeal (TS) nerve using 3D printed guides were demonstrated (Fig. 1e) (Lissandrello et al. 2017).

Autonomic nerve fascicles have a small diameter and are insulated with perineurium. In addition to the biocompatibility of the neural interface, these limitations make it difficult to create a high resolution of recording and stimulation. Therefore, the intrafascicular electrode, a way of penetrating into the nerve, was developed for a high SNR recording. One of the recent intrafascicular electrodes being studied was Carbon fiber on polyimide ultra-microelectrodes. This electrode was fabricated with a flexible carbon fiber and flexible polyimide, and an alignment tool. In addition, to improve poor charge injection capacity, which is a disadvantage of small diameter carbon fiber, 100 nm iridium oxide film was electrodeposited. The fabricated electrode recorded the high quality of nerve signals from a TS nerve of 125 μm (Fig. 1f) (Gillis et al. 2018). Furthermore, an intrafascicular carbon nanotube yarn (CNTY) interface fabricated using a carbon nanotube fabrication technique was demonstrated. A new injection method based on microneurography technology was developed to insert these flexible materials into the vagus nerve. This electrode showed a high signal-to-noise ratio (> 10 dB) and was capable of chronic implantation for up to 16 weeks (Fig. 1g) (McCallum et al. 2017). An example of how to use this autonomic nerve interface is a closed-loop bladder neuroprosthetic implemented by implanting microchannel electrodes into the L6 dorsal rootles connected to the pudendal nerve (Fig. 1h) (Chew et al. 2013).

### Wireless neural interface

**Fig. 2:**
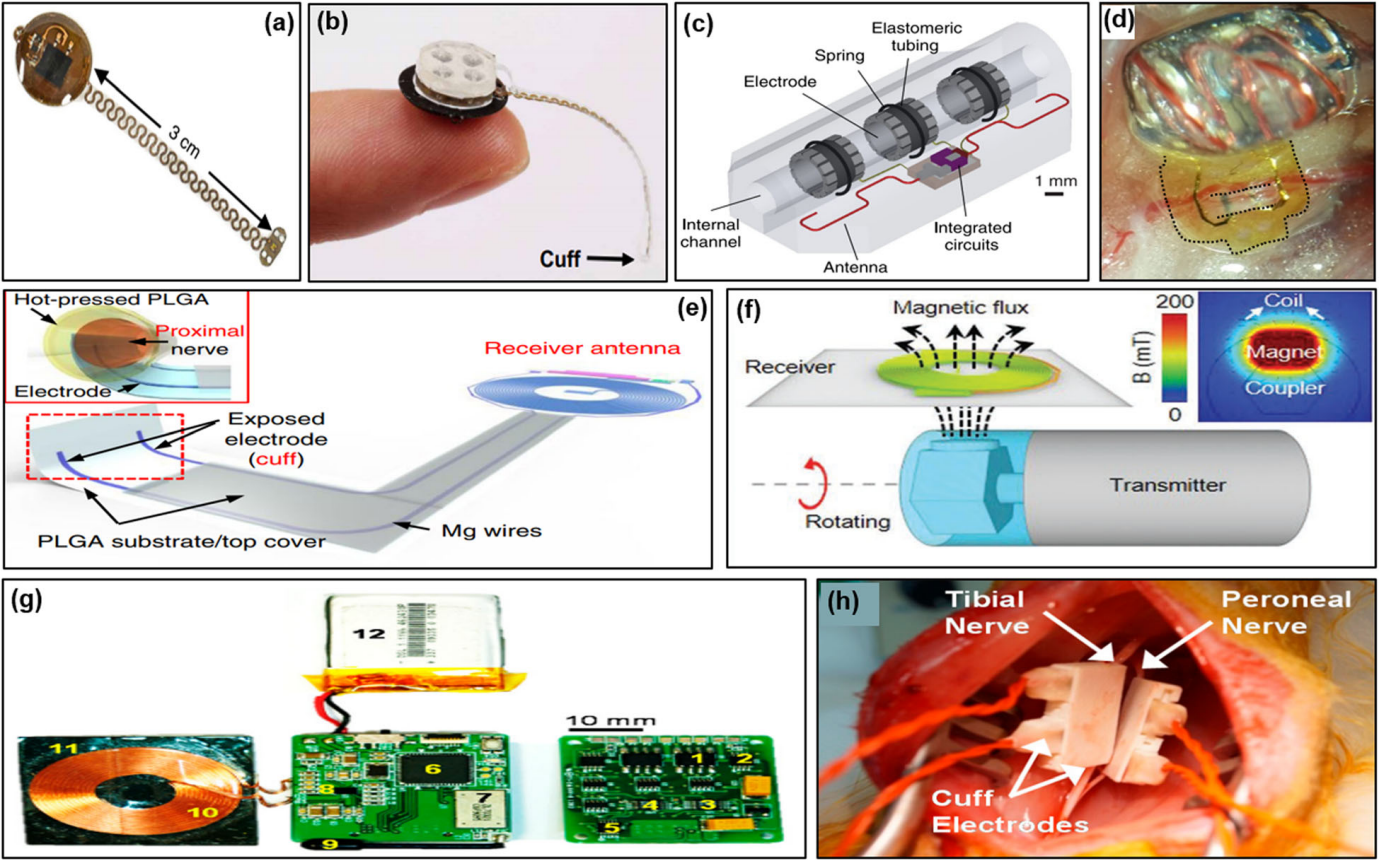
Wireless neural interfaces **a** Wireless, battery-free, fully implantable multimodal and multisite pacemaker for small animal model. **b** Wireless neural interface for wireless optogenetic and pharmacological neuromodulation of peripheral nerves. **c** Mid-field range wireless neural interface for vagus nerve. **d** Wireless neural clip interface for bladder modulation. **e** Wireless bioresorbable neural interface. **f** Bioresorbable magnetically coupled system. **g**, **h** Implantable wireless neural interface system for stimulation and recording

Wireless power transmission technologies for active neural interfaces such as wirelessly stimulating nerve or wirelessly recording neural signals are required. Since the autonomic nerve is very small in size, and is located deep inside the body, more efficient wireless communication technologies are required. Power transmission technology through wireless communication technology in the near field area was used. The most commonly used technology in this area is Near Field Communication (NFC). For instance, a pacemaker was developed that is operated by transmitting power through wireless communication in the 13.56 MHz, NFC frequency band (Fig. 2a) (Gutruf et al. 2019). In addition, NFC was used to supply power to the microscale inorganic light-emitting diode through wireless power transmission for optogenetic applications to stimulate the sciatic nerve of mouse (Fig. 2b) (Zhang et al. 2019). However, in the above study, the animals were confined in a customized power transfer chamber to transmit the power, due to the short transmission distance of NFC. The power transfer efficiency (PTE) of NFC is higher than in mid-field wireless powering, but the relatively huge size of the receiving antenna can be limited to the implant. Reducing the size of the antenna also leads to an increase in the optimum frequency for operation (Yang et al. 2020). For this reason, a wireless neural interface with a high PTE in the midfield area was developed by reducing the size of the antenna. This wireless neural interface achieved power transfer efficiencies from 8% with 1 cm coupling distance under the tissue (Fig. 2c) (Tanabe et al. 2017). Another midfield range neural interface for wireless stimulation, an active neural clip was demonstrated to modulate bladder function. Active neural clip was made of 16 μm thin and flexible polyimide materials. It was integrated with wireless components with a small coil (dia: 2 mm). This interface was successfully implanted on the pelvic nerve (250–300 um), and the bladder pressure measurement was confirmed that it was wirelessly stimulated in the midfield range (~ 1.5 GHz) (Fig. 2d) (Lee et al. 2017a).

Wireless neural interfaces eliminate the need for battery replacement and continuously transfer the energy unless the interface and components are broken. However, there are some therapeutic applications that require neural stimulation only for a certain period of times. In this case, there are still burdens of removing the interface after the treatment. To implement such a requirement, a wireless neural interface made of biodegradable materials was developed. It consisted of a bioresorbable antenna and a bioresorbable electrode using Mg and PLGA. The developed neural interface stimulated the rat’s sciatic nerve through power transmission at 5 MHz, and it had little parasitic absorption by biological tissues (Fig. 2e) (Koo et al. 2018).

Most of the above wireless neural interfaces required a micro controller or a circuit to realize stimulation pulses such as monophasic and biphasic pulses that were used for neural stimulation (Gutruf et al. 2019; Koo et al. 2018; Zhang et al. 2019). It is well known that a biphasic pulse (a charge-balanced waveform) is preferred for neural stimulation since it avoids damage to electrodes and surrounding tissue (Brocker and Grill 2013; Cogan 2008). A study on direct biphasic pulse stimulation through the change of magnetic field using magnetic coupling was reported without using a microcontroller or circuit (Fig. 2f) (Guo et al. 2019).

For wireless recording of neural interfaces, it is required to implant the battery and the wireless communication module inside or outside the body. A reconfigurable wireless neural interface system using commercial off-the-shelf (COTS) components was developed. This wireless neural interface successfully recorded neural signals and stimulated the tibial and peroneal nerves while communicating with the external device (Fig. 2g, h) (Shon et al. 2018). These systems demonstrate the positive probability of implementing closed-loop control devices like feedback control for prosthesis.

### Energy harvesting for neural interface

Conventional stimulators require electronic circuits and components to generate electrical pulses required for therapeutic responses. Advanced batteries which are smaller and have prolonged lifetime, as well as wireless system that transfers energy efficiently into bioelectronics located deeply inside body are constantly suggested and studied, but novel concepts are required for future direction of bioelectronic medicines. Recently, the concept of converting mechanical human body energy into electrical power by various mechanism has been explored as an alternative way to support the operation of implantable medical devices as well as to directly use electrical energy generated for stimulating nerves (Liu et al. 2019 Adv Func Mater; Hinchet et al. 2019 Science; Wang et al. 2019b Nano Energy; Wang et al. 2019a Adv Sci; He et al. 2019 Adv Sci). Triboelectric nanogenerators (TENGs) have recently been demonstrated as a promising technology providing advantages of lower cost, a wider range of material choice (flexible, biocompatible, and biodegradable), easy fabrication, and high power output (Jiang et al. 2020 Adv Mater; Liu et al. 2020 Nano Energy; Wang et al. 2020a Nano Energy; Dong et al. 2020 ACS Nano; Wen et al. 2020 Adv Sci; Zhu et al. 2020 Nano Energy; Shi et al. 2019 Nano Energy).

**Fig. 3:**
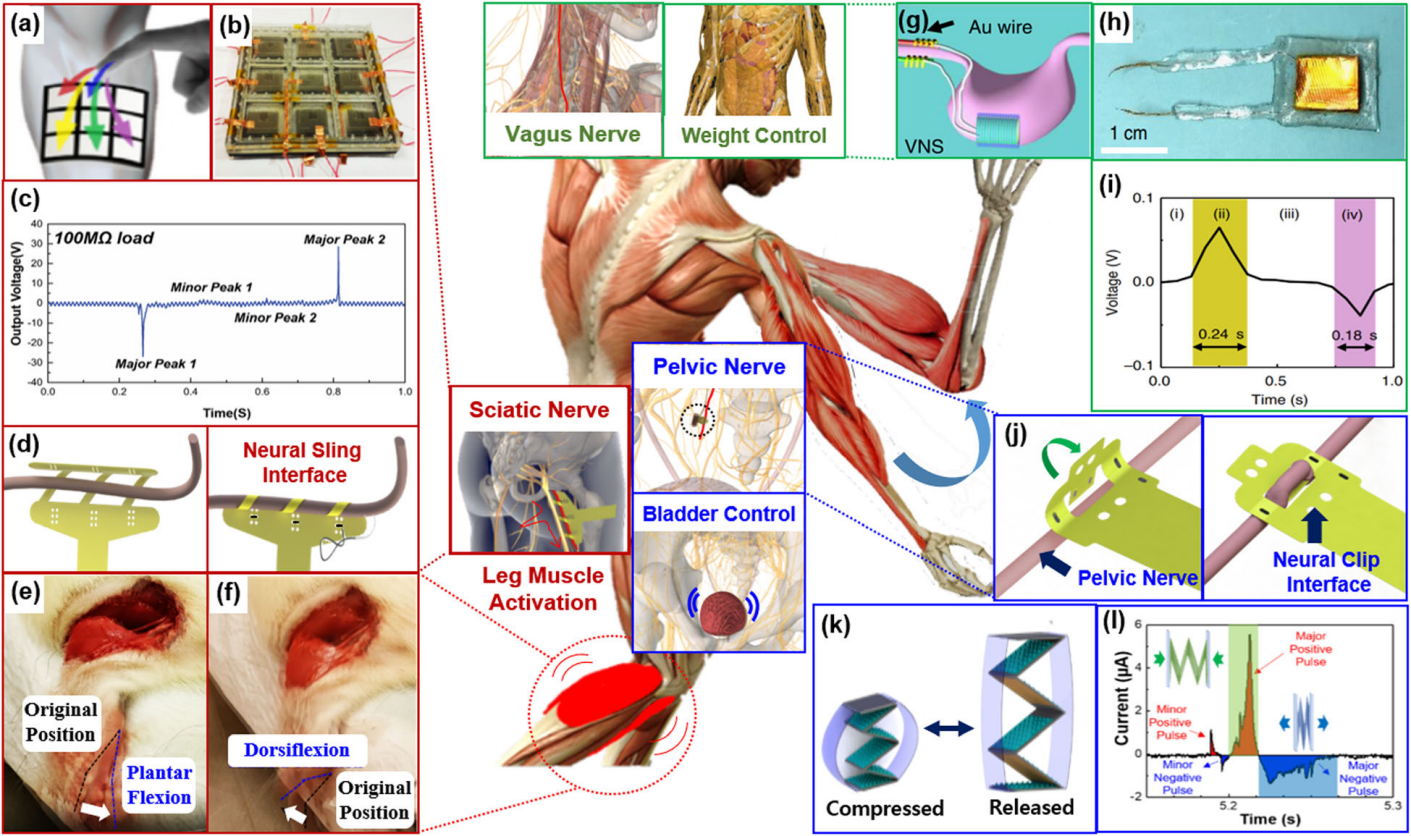
A conceptual diagram of a self-sustainable neural interface. **a** Schematic diagram of wearable and multi-layered triboelectric nanogenerators (TENGs). The picture of the fabricated TENGs. **c** The output signals generated by the TENGs. **d** Schematic diagram of implanting sling interface on a nerve. **e** The plantar flexion and **f** dorsiflexion of a rat evoked by selective stimulation using the sling interface combined with TENGs. **g** Schematic diagram of vagus nerve stimulation using TENGs and **h** the picture of the TENGs. **i** The output signals generated by the TENGs. **j** Schematic diagram of implanting neural clip interface on a nerve. **k** Schematic diagram of fabricated TENGs and **l** the charge-balanced biphasic signals generated by the TENGs

Recent interesting studies indicate that electrical energy generated by TENGs could be used for direct nerve stimulation by combining with neural interface without electronic circuits (Lee et al. 2017a, b; He et al. 2019 Adv Sci). Multiarray-type TENGs were demonstrated for direct and selective nerve stimulation by combining with flexible sling interfaces (Fig. 3a-d) (Lee et al. 2018a). During pressing and releasing the arrays, negative and positive pulses were generated, thereby stimulating nerve twice (Fig. 3c). The implanted sling interface on a sciatic nerve in rats provided six multiple electrode contacts helically around the nerve, so the selective activations of leg muscles (plantar flexion and dorsiflexion) were achieved by selectively stimulating the sciatic nerve using the multiarray TENGs (Fig. 3d-f).

VNS to treat obesity was demonstrated using biocompatible TENGs (Yao et al. 2018 Nat Comm). The TENGs implanted during 12 weeks on stomach generates electrical signals during the stomach motion, and the electrical pulses generated were delivered via a neural interface implanted on a vagus nerve for VNS (Fig. 3g-h). The output pulses showed biphasic, but long duration and long interpulse interval. These stimulation pulses could stimulate a nerve twice, which needs to improve for precise and reliable stimulation of autonomic nerves. More recently, neural clip interfaces, which enabled to implant on the pelvic nerve for bladder control, combined with an improved TENG which generates asymmetric biphasic pulses (Lee et al. 2019). This asymmetric biphasic pulse, which was a charge-balanced biphasic pulse, reliably stimulated the pelvic nerve thereby inducing urination in rats.

Various mechanical movements from muscles or organs in the body can be used for the energy to stimulate nerves in the future. Recently, implantable energy harvesters are being developed with high performance and various functions. Along with this cutting-edge technology, advanced energy harvesters should be developed that use several forms of biomechanical energy to generate appropriate neurostimulation waveforms. Furthermore, toward clinical applications, the size and the lifetime of the energy harvesters would be critical issues.

### Other modalities

Electrical stimulation, which is a conventional way by delivering electrical currents to neural tissue, has been used for activating nerve fibers. However, precisely stimulating an individual nerve fiber is limited since the current spreads out nearby tissue that are not targeted. For the autonomic nervous systems that have a significant effect on bodily functions, this can be critical issue since unintentional stimuli to off-target leads to serious side effects. Also, the electrical stimulation is mainly based on the contact-mode mechanism where conducting materials (mainly metals) directly contact with tissue so that electrical current can deliver through tissue. This is directly relevant to the safety of the tissue and the reliability of the electrode, especially for long-term use. For instance, implants cause immune responses to form scar tissue covering electrode. This reduces the performance of stimulation or recording. Accordingly, nowadays, many studies of non-contact modulation dramatically have been reported such as using optic, magnetic, and ultrasound in a non-invasive manner.

#### Ultrasound stimulation

**Fig. 4:**
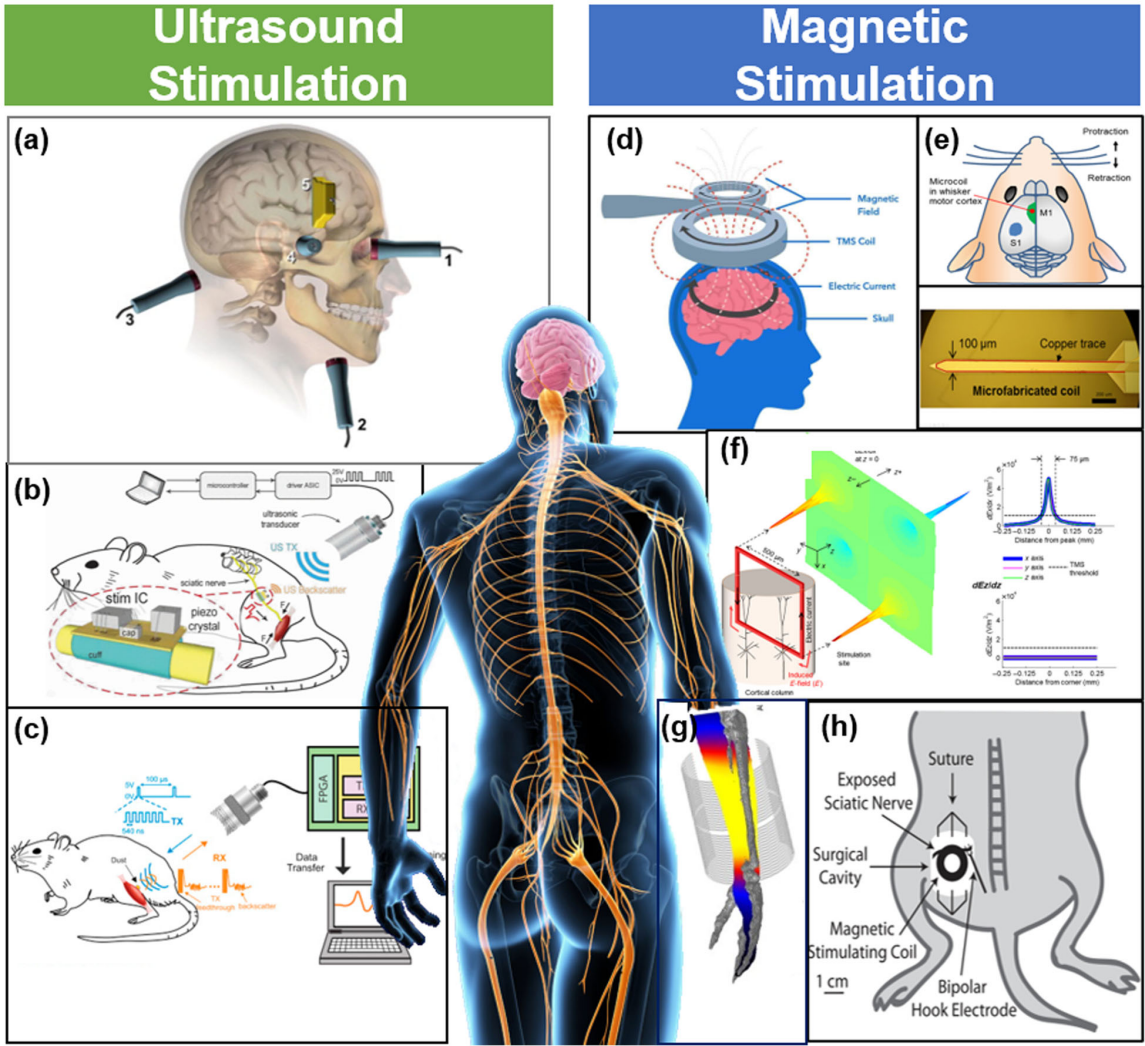
Ultrasonic stimulation (left) and magnetic stimulation (right) in non-invasive manner. **a** Schematic figure of focused ultrasound. **b** Schematic figure of In vivo experimental setup for induced EMG recording in rats using ultrasound. **c** Schematic figure of StimDust system to stimulate a sciatic nerve in a rat. **d** Schematic figure diagram of transcranial magnetic stimulation (TMS); A magnetic field that occurs in a coil above the head induces an eddy current that stimulates the nerve. **e-f** Schematic of the microfabricated coil (bottom). Coils were inserted into the whisker motor cortex (upper). Measurement of electric field gradients in coil using simulation. Surface plot of the electric field gradients in the x direction (left). Two- dimensional profile of the gradients in the vertical and horizontal directions (right). **g** Illustration of simulation result indicating the threshold of peripheral nerve stimulation during MRI measurement. **h** Illustration of magnetic stimulation of a sciatic nerve in a rat. The coil was positioned onto the exposed sciatic nerve

One of the new ways to stimulate the human nervous system is ultrasound stimulation (US). The mechanism of US has been generally suggested in two ways. The first mechanism is the opening of the ion channel by the stretch of membrane induced by the cavitation force, and the second one is the depolarization through sonoporation caused by the same cavitation mechanism (Wright et al. 2017). Other mechanisms were also suggested such as thermal effects, but more studies are required. There are different modes of ultrasound stimulation. For a brain in the central nervous system (CNS), Transcranial Ultrasound (TUS), which is non-invasively stimulating the neuron in targeted brain regions by using pulsed ultrasound was demonstrated as a treatment method of neurological disorders. For example, low-intensity transcranial ultrasound stimulation significantly decreased parkinsonian-related activity in mice by stimulating the motor cortex. (Wang et al. 2020b). TUS also showed the effect on pain control, and it was effective in improving patients’ mood (Hameroff et al. 2013) (Fig. 4a). Furthermore, research using US was conducted in the Peripheral Nerve System (PNS). Neural stimulation using Focused Ultrasound (FUS) stimulated nerves with a spatial resolution of 100 μm by concentrating energy in a limited area (Kubanek 2018). It was successfully evoked leg muscle activations through the stimulation of the sciatic nerve in mice, which demonstrates that it would be a promising alternative method in peripheral nerve stimulation as a noninvasive manner (Downs et al. 2018).

The US can be used as an energy transfer source rather than as a stimulation energy source, which enables to build a wireless system. An mm-sized stimulator with piezo crystals and single electrode was implanted on the sciatic nerve of the rat to achieve wireless stimulation using US (Johnson et al. 2018) (Fig. 4b). And it achieved the highest efficiency (82%) at the smallest volume (6.5 mm^3^) and mass (10 mg). Also, the communication system using ultrasonic backscatter enabled wireless recording of physiological signals acquired in vivo. According to the study, the EMG signal generated from the gastrocnemius muscle of rat was successfully measured by using a neural dust that was assembled on a flexible PCB consisting of a piezoelectric crystal, transistor, and recording electrodes. (Seo et al. 2016) (Fig. 4c). Neural dust could become even more effective with a few adjustments. For example, a flat, low-profile piezo transducer would enable a smaller implant device.

#### Magnetic stimulation

Another method for non-invasive modulating the nervous system is magnetic stimulation. Magnetic stimulation on neural tissue has been already well known as transcranial magnetic stimulation (TMS), and it showed remarkable therapeutic effects in clinical applications (Fig. 4d). The Food and Drug Administration (FDA) approved TMS for the treatments of some disorders such as depression (Horvath et al. 2010). The research results on the stability of TMS are positive (Anand and Hotson 2002). The general mechanism of magnetic stimulation on neural tissues is by eddy current formed in the nerve by the alternating (AC) magnetic field. (Öberg 1973). TMS has larger size coils to generate an AC magnetic field large enough to induce neural activation and to focus on specific regions in a brain. Accordingly, it requires larger energy consumption than electric stimulation and causes heating and vibration issues during the stimulation. However, there are huge advantages of magnetic stimulation such as non-invasive method and no attenuation of the field inside body, and the field-based stimulation enabling multiple stimulations. Therefore, so many researchers focus on overcoming the limitations. For instance, the micro-sized implantable coils were developed to reduce the energy consumption and selectivity (Lee et al. 2016) (Fig. 4e). According to Lee, it succeeded in stimulating the motor cortex of a rat using a micro-sized coil with less energy consumption than TMS. The TMS using millimeter-sized coils was targeted at the monkey’s motor cortex. As a result, approximately 75 kW of power was needed to trigger the movement. And in experiments with micro coils, the coils were inserted into the motor cortex of mice. Then, it induced the movement with a power of 10 mW. Both coils were required higher power than the power used in the deep brain stimulation (DBS) using electrical stimulation. However, the micro coils reduced the power consumption significantly by approaching to targeted tissue enhancing spatial resolution. These conclusions were drawn through indirect comparisons, not direct comparisons between TMS and micro coils, due to differences in the size and the shape of coils, subjects of experiments, etc. In the magnetic stimulation used in the micro coil, the characteristic of the coil showed the asymmetric profile of the induced potential gradient (Lee et al. 2016) (Fig. 4f), thereby showing the promising possibility for the selective neuron activation. Furthermore, the characteristics of different coil shapes, and the effect of the coils on neural stimulation were demonstrated (Lee et al. 2018b). The in vitro experiment was conducted with coronal brain slices of mouse primary visual cortex (V1) to compare the performance of the coils of V and W shaped design. The direct comparison of the performance of the coils were performed under the same condition such as physiological state and stimulation parameters. The results showed that the W-shaped coil could perform the most selective stimulation. However, the W-shaped coil enhanced spatial selectivity while it reduced stimulus intensity (stimulation thresholds for V and W shaped coils are 13.07 mA and 15.08 mA at 5 kHz sinusoidal waveform). In PNS, various studies were conducted. For instance, a study was conducted to measure the PNS threshold through simulation to avoid peripheral nerve stimulation during MRI measurement. This is because the magnetic field changes in the kilohertz frequency range used in MRI are sufficient to produce PNS in the human body (Davids et al. 2017) (Fig. 4g). Another study of magnetic stimulation in PNS was demonstrated with different coils. In this paper, centimeter-sized coils were used for sciatic nerve stimulation in rats, requiring energy of 20 J (Kagan et al. 2016) (Fig. 4h).

## Conclusions and future perspectives

The next generation of neural interface for bioelectronic medicine should deal with interfaces, functions, systems. Interfaces must reliably interact with autonomic nerves for long period as well as have functions with high resolution of stimulation and recording. Multi-disciplinary research such as micro/nano engineering, materials science/engineering is required. Furthermore, to demonstrate the performance of recording and stimulation in the interface, various forms of active neural interfaces, which combine the interface with an amplifier for recording or with a stimulator for stimulation, should be developed with initial design of the interface. This is directly relevant to the energy issue. For stimulation, electrical, magnetic, ultrasound stimulations were investigated, but optical stimulation was not covered. Electrical stimulation was typically demonstrated, and the results of wireless transmission with electrical circuits and wireless antenna for stimulation and recording were demonstrated. However, it is true that the minimized interface and systems required for autonomic nerves seem to have many difficulties in implementing active neural interfaces for bioelectronic medicine. Therefore, novel ways for stimulation are receiving great attention now. Ultrasound stimulation and magnetic stimulation have shown positive results nowadays. Those are still in their early stages but are developing dramatically. A self-sustainable interface without a battery and electronic circuits was also demonstrated with triboelectric nanogenerators. The vagus nerve and the pelvic nerve stimulation were recently demonstrated, and those showed promising results. The TENG provided charge-balanced biphasic pulses without additional electric circuits, which have benefits in minimizing the system and intuitive direct stimulation. This self-sustainable interface concept would be a good solution where many interfaces are implanted on different nerves requiring different stimulation situations. It will provide continuous energy for neurostimulation while moving the body. For this, advanced mechano-stimulators, which convert various mechanical energies originated from our body to the stimulation pulses, should be preferentially developed. Also, advanced neural interface should be developed together, and the system that maintains appropriate stimulation will be required. This is also in the early stages, but we expect the self-sustainable interface would be promising as a new treatment technology that can be applied to the autonomic nervous system and the peripheral nervous systems.

## Data Availability

References are listed in the references section.

## Abbreviations

PNS: Peripheral nervous system
ANS: Autonomic nervous system
CNS: Central Nervous System
SNR: Signal-to-noise ratio
CN X: Tenth cranial nerve
VNS: Vagus nerve stimulation
FDA: Food and Drug Administration
SMP: Shape memory polymers
PN: Pelvic nerve
VN: Vagus nerve
TS: Tracheosyringeal
CNTY: Carbon nanotube yarn
NFC: Near Field Communication
TENG: Triboelectric nanogenerators
US: Ultrasound stimulation
TUS: Transcranial Ultrasound
FUS: Focused Ultrasound
TMS: Transcranial magnetic stimulation
AC: Alternating
